# Prognostic value of Th17 cells in acute leukemia

**DOI:** 10.1007/s12032-013-0732-3

**Published:** 2013-10-02

**Authors:** Nashwa Khairat Abousamra, Manal Salah El-Din, Randah Helal

**Affiliations:** 1 Department of Clinical Pathology, Hematology Unit, Faculty of Medicine (35516), Mansoura University, Mansoura, Egypt; 2 Department of Medical Oncology, Oncology Center, Mansoura University, Mansoura, Egypt; 3 Department of Public Health and Community Medicine, Faculty of Medicine, Mansoura University, Mansoura, Egypt

**Keywords:** Th17 cells, Acute leukemia, IL-17, IL-21

## Abstract

Th17 cells and their effector cytokines have emerged as important mediators in inflammatory and autoimmune diseases and serve as an ambitious field in current immunology research. Recent studies suggest a potential impact of Th17 cells on solid tumors but relatively little is known about their contribution in hematological malignancies. The current study was designed to investigate the possible involvement and clinical significance of circulating Th17 cells in acute leukemia. Flow cytometry was used to analyze percentages of Th17 cells in peripheral blood mononuclear cells from 93 acute leukemia patients (ALL, *n* = 30; AML, *n* = 63) and 40 healthy volunteers. Serum levels of IL-17 and IL-21 were measured using enzyme-linked immunosorbent assay. Circulating Th17 cells were increased in patients with acute leukemia (2.88 ± 0.65 % and 2.90 ± 0.57 % in ALL and AML patients, respectively) and were significantly higher than in healthy controls (1.10 ± 0.28 %; *P* = 0.001). Furthermore, pretreatment Th17 cells were reduced significantly in patients who achieved complete remission after induction therapy (2.25 ± 0.44 % and 1.63 ± 0.27 % in ALL and AML patients, respectively, *P* < 0.0001). Serum levels of IL-17 and IL-21 were significantly elevated in acute leukemia patients. Kaplan–Meier curves revealed a significantly longer overall survival in patients with high Th17 levels (*P* = 0.029 and *P* = 0.027 for ALL and AML, respectively). In the multivariate analysis, Th17 cells retained statistical significance for overall survival in patients with ALL (OR 0.331; *P* = 0.043) and AML (OR 0.489; *P* = 0.032). These results strongly suggest Th17 cells as a powerful new prognostic determinant which could serve as a potential therapeutic target to modulate anti-tumor response in acute leukemia patients.

## Introduction

Adaptive immunity plays a crucial role in tumor immunosurveillance [1, 2]. It has been shown that tumor-infiltrating effector T cells are associated with improved prognoses in multiple human cancers [3–5], whereas tumor-infiltrating regulatory T cells (T reg) are negatively associated with patients’ outcome [5, 6].

T helper 17 (Th17) cells are newly identified effector CD4+ cells [7]. Their discovery has resulted in an explosion of immunological research, and it is now widely accepted that the Th17 subset is an independent lineage of Th cells based on their unique cytokine profile, transcriptional regulation, and biological function [8, 9]. They release their signature cytokine IL17A together with IL17F, IL21, IL22, and IL26 [10]. The identification of Th17 cells not only changes the classical Th1/Th2 paradigm of Th cell differentiation but also markedly facilitates our understanding of human immunity under both physiological and pathological conditions [9].

It has been suggested that Th17 cells were increased in patients with solid tumors [11] but little is known about Th17 cells in hematological malignancies.

IL-17 has pro- and anti-tumor actions [12]. It was suggested that endogenous IL-17 positively impacts on tumor immunity [13]. Th17 cells have also been shown to produce IL-21, a cytokine that can amplify the differentiation of Th17 cells in an autocrine manner as well as control T cell-dependent humoral responses [14, 15]. Increasing evidence suggests an involvement of IL-21 in hematopoietic malignancies [16].

Acute leukemias represent ideal models to assess the impact of cancer on the host immune system as the disease is widely disseminated, so that immune cells in the peripheral blood are in close proximity to the tumor cells. Failure to recognize and eradicate leukemia cells may partly be a result of insufficient immunological activation. Despite the few studies focusing on Th17 cells in acute leukemia, there are controversies with regard to their results [17, 18]. In this study, we determined the prevalence of circulating Th17 cells and serum concentration of their related cytokines IL-17 and IL-21 in adult acute leukemia patients to evaluate their potential impact on disease outcome.

## Subjects and methods

### Patients

Between May 2010 and December 2012, peripheral blood samples were obtained with informed consent and Institutional Review Board approval from 93 newly diagnosed patients with acute leukemia (63 patients with AML and 30 patients with ALL) and 40 age-matched healthy volunteers. Diagnosis and classification were based on WHO classification [19]. Data describing the study subjects are summarized in Table 1. Response was evaluated after induction therapy in 87/93 patients. Two patients (one patient with ALL and the other with AML) received only supportive care while four patients (one patient with ALL and three patients with AML) died during induction therapy before hematological recovery and were excluded from the response analysis. Patients with T-ALL were excluded from this study because of their low incidence and the induced immune tolerance hypothesized in those patients [20].

**Table 1:** Subjects’ characteristics

Parameters	ALL (*n* = 30)		AML (*n* = 63)		Controls (*n* = 40)
Gender					
Male		18 (60 %)		33 (52.3 %)	23 (57.5 %)
Female		12 (40 %)		30 (47.6 %)	17 (42.5 %)
Age					
Median (range)		31.5 (19–59)		48 (23–63)	42 (20–61)
Remission					
Yes	25/28	(89.3 %)	37/59	(62.7 %)	
No	3/28	(10.7 %)	22/59	(37.3 %)	
Cytogenetic abnormalities	Missing	10 (33.3 %)	Favorable^a^	17 (27 %)	
	Ph −ve	17 (56.7 %)	Intermediate^b^	26 (41.3 %)	
	Ph +ve	3 (10 %)	Adverse^c^	20 (31.7 %)	

^a^Favorable: *t*(15; 17)(q22; q21), *t*(8; 21)(q22; q22), inv(16)(p13; q22)/*t*(16; 16)(p13; q22)

^b^Intermediate: entities not classified as favorable or adverse

^c^Adverse: −5/5q−, −7/7q−, abn(11q23), or complex karyotype with three or more cytogenetic abnormalities

### Main reagents

The following anti-human monoclonal antibodies were used: fluorescein isothiocyanate (FITC)-labeled anti-CD4, R-phycoerythrin-cyanine 5 (RPE-CY5)-labeled anti-IL-17A, phycoerythrin (PE)-labeled anti-CD3 (eBioscience, San Diego, USA), and their appropriate isotype controls (Dakocytoformation, Denmark). Fixation and permeabilization of cells were done using Intraprep kits reagent (immunotech, Beckman Coulter, Marseille, France). Phorbol myristate acetate (PMA) and ionomycin were purchased from Sigma, USA. Human IL-17 and IL-21 ELISA kits were purchased from Bender Medsystems, Austria.

### Flow cytometric analysis

Peripheral blood mononuclear cells (PBMCs) were isolated by standard Ficoll-Hypaque density centrifugation of heparinized peripheral blood from healthy control subjects and patients before and after induction chemotherapy. PBMCs were cultured in RPMI 1,640 medium that contained 100 U/mL penicillin, 100 U/mL streptomycin, and 10 % fetal bovine serum. Cell density was adjusted to 2 × 10^6 ^cells/mL. Cells were stimulated by adding 50 ng/mL PMA, 1 μg/mL ionomycin, and 10 μg/mL BFA to the medium for 5 h at 37 °C under 5 % CO2. Stimulated cells were centrifuged at 1,200*g* for 7 min at 10 °C, washed, and then surface stained by incubating cells (1–2 × 10^6^/mL) for 30 min at room temperature in the dark with fluorescence-labeled anti-CD3 and anti-CD4. For intracellular staining, cells were subsequently treated with cell fixation and permeabilization reagents following the manufacturer’s instructions and then incubated with a fluorescence-labeled anti-IL-17A for 30 min at 4 °C. Cells were resuspended in PBS and analyzed using the EPICS XL flow cytometer (Coulter Electronic, Fl, USA). Appropriate isotypic controls were used to determine specific binding for each fluorescent channel. After gating on CD3+ CD4+ cells, Th17 cells were defined as the percentage of IL-17+ cells.

### Enzyme-linked immunosorbent assay (ELISA)

The serum of patients and healthy volunteers was analyzed for the Th17-related cytokines IL-17 and IL-21 using ELISA, following the manufacturer’s instructions. All samples were measured in duplicate.

### Statistical analysis

Data were analyzed using SPSS, version 16. K–S test was used to test the normality of data. Descriptive analysis of the collected data was done with calculation of frequencies, median, and range. In normally distributed variables, student’s *t* test, paired *t* test, and one-way ANOVA were used to compare the means between different groups, as appropriate. Correlation between Th17 and other parameters was examined by Spearman’s correlation coefficient. Overall survival was measured from the time of diagnosis to death or last observation. It was calculated and represented by Kaplan–Meier plots and the log-rank test, which was also used to evaluate the possible associations between OS and each risk factor singly. All the parameters of significant value in univariate analysis were entered in a stepwise multivariate Cox proportional hazard model. *P* ≤ 0.05 was chosen as the level of statistical significance.

## Results

### Increased circulating Th17 cells in acute leukemia patients

**Fig. 1:**
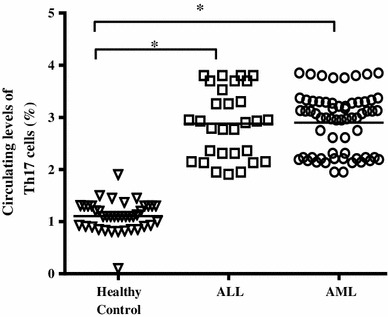
Increased levels of circulating Th17 cells in untreated patients with ALL and AML compared to healthy controls

We investigated levels of circulating Th17 cells in untreated AML and ALL patients as well as in healthy controls. Representative plots showed that circulating Th17 cells were increased in untreated patients (2.88 ± 0.65 % and 2.90 ± 0.57 % in ALL and AML patients, respectively) compared to healthy volunteers (1.10 ± 0.28 %), where the difference was statistically significant (*P* < 0.001) (Fig. 1). However, difference in circulating Th17 cells between ALL and AML patients before treatment was not statistically significant (*P* = 0.862).

### Level of circulating Th17 cells differs according to therapeutic response in acute leukemia patients

**Fig. 2:**
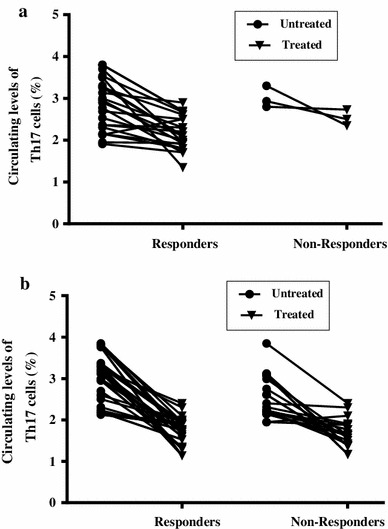
Comparison of circulating Th17 cells in ALL (**a**) and AML (**b**) patients before and after chemotherapy. Th17 cells were reduced significantly in responders compared to their levels before treatment

**Fig. 3:**
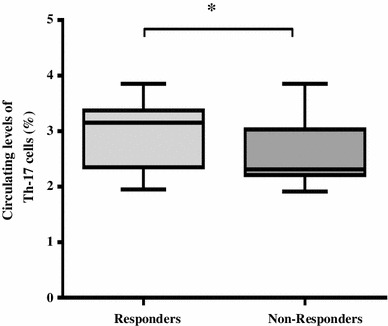
Increased pretreatment levels of circulating T helper-17(Th17) cells in responders (*n* = 62) compared to non-responders (*n* = 25) of acute leukemia patients. All results are presented as mean (*horizontal line*). Minimum and maximum (*boxes*) and variation range (*whiskers*)

Sixty-two patients responded to treatment and achieved complete remission (CR) following induction therapy (25 ALL and 37 AML patients) while 25 patients were non-responders. As shown in Fig. 2, the percentages of circulating Th17 cells were reduced significantly in ALL (Fig. 2a) and AML (Fig. 2b) responders (2.25 ± 0.44 %, 1.63 ± 0.27 %, respectively) compared with the same patients before treatment (2.88 ± 0.65 %, 3.09 ± 0.55 %). Interestingly, significantly higher pretreatment levels of Th17 cells were observed in responders (3.032 ± 0.58 %) when compared to non-responders (2.53 ± 0.49 %) in the whole group of acute leukemia patients (*P* < 0.0001) (Fig. 3). Moreover, low levels of Th17 cells (1.95 and 2.23 %) were observed in the two patients who were not eligible for chemotherapy and received supportive care only.

### Increased serum concentrations of IL-17 and IL-21 in acute leukemia patients

The IL-17 and IL-21 serum concentrations showed statistically significant higher levels in acute leukemia patients compared to healthy volunteers (*P* < 0.0001 for IL-17 and IL-21, respectively, for both ALL and AML) (Table 2). There was no significant difference regarding IL-17 concentration between ALL and AML patients. However, ALL patients showed statistically significant higher serum concentrations of IL-21 than AML patients (*P* = 0.002). Additionally, serum concentrations of IL-17 and IL-21 showed positive correlation with circulating Th17 cells in both ALL (*r* = 0.768, *P* < 0.0001, *r* = 0.548, *P* < 0.0001 for IL-17 and IL-21, respectively) and AML patients (*r* = 0.897, *P* < 0.0001, *r* = 0.765, *P* < 0.0001 for IL-17 and IL-21, respectively).

**Table 2:** Concentration of IL-17 and IL-21 serum levels among acute leukemia patients compared to healthy controls

	Healthy control (*n* = 40)	ALL (*n* = 30)	AML (*n* = 63)	One-way ANOVA	Post hoc tests
IL-17 (pg/mL)	12.89 ± 4.9	43.2 ± 13.5	40.78 ± 15.30	*P* = 0.0001	(1) versus (2) = 0.0001 (1) versus (3) = 0.0001 (2) versus (3) = 0.393
IL-21(pg/mL)	252.8 ± 45.1	1257.5 ± 718.2	944.3 ± 394.3	*P* = 0.0001	(1) versus (2) = 0.0001 (1) versus (3) = 0.0001 (2) versus (3) = 0.002

### Circulating Th17 cells and overall survival (OS) in acute leukemia patients

**Fig. 4:**
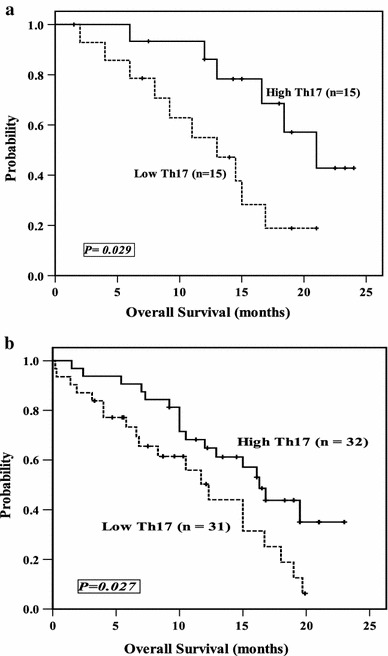
Kaplan–Meier plot comparing OS based on the median level of circulating Th17 cells in ALL (**a**) and AML patients (**b**)

Patients were dichotomized into two groups according to the median level of circulating Th17 cells (median = 2.91 and 2.99 % for ALL and AML, respectively). Group 1 included patients with Th17 cells above the median level (Th17^High^; *n* = 15 and 32 ALL and AML patients, respectively), and group 2 included patients with Th17 cells lower than the median level (Th17^low^; *n* = 15 and 31 ALL and AML patients, respectively). Survival analysis was performed and, as depicted in Fig. 4a, b, in group 1 patients, the median overall survival was longer than in group 2 (16.6 vs. 11 months in ALL, *P* = 0.029; 13.9 vs. 8.3 months in AML, *P* = 0.027).

### Circulating Th17 cells as a significant independent positive prognostic factor in acute leukemia

Despite the heterogeneity of our patients, univariate analysis identified age (*P* = 0.008), WBCs (*P* = 0.034), IL-21 (*P* = 0.005), IL-17 (*P* = 0.014), in addition to Th17 level (*P* = 0.029) as predictors for OS in patients with ALL. In patients with AML, Th17 level (*P* = 0.027), WBCs (*P* = 0.041), age (*P* = 0.038), adverse cytogenetic abnormalities (*P* = 0.001) and IL-17 (*P* = 0.035) were all significant factors. When these prognostic factors were considered in a multivariate analysis, Th17cells and IL-17 serum level merged as significant independent positive prognostic factors for OS. Age and IL-21 maintained their discriminating power as poor prognostic factors in ALL. In AML, adverse cytogenetic abnormalities remained significant negative predictor for survival (Table 3).

**Table 3:** Multivariate Cox proportional hazard model of overall survival in acute leukemia patients

Variables	ALL				Variables	AML			
	OR	95 % CI		*P*		OR	95 % CI		*P*
		Lower	Upper				Lower	Upper	
High Th17 (%)	0.273	0.09	0.829	0.02	High Th17 (%)	0.489	0.254	0.941	0.032
High IL-17 (pg/mL)	0.406	0.112	1.467	0.16	High IL-17 (pg/mL)	0.522	0.263	1.034	0.062
WBCs (>50 × 10^9^/L)	2.916	0.821	10.353	0.09	WBCs (>50 × 10^9^/L)	1.922	0.990	3.733	0.054
Age >35 (years)	4.001	1.267	12.63	0.01	Adverse cytogenetics	2.711	1.393	5.275	0.003

## Discussion

The magnitude of the data regarding Th17 cells in experimental tumor models and cancer patients suggests that the role of Th17 cells in tumor immunity is highly complex, and it remains controversial whether these cells promote tumor growth or regulate anti-tumor responses [21]. Further complicating the matter are occasionally conflicting results of studies and contradictory data about the role of Th17 in patients with acute leukemia [17, 18, 22].

In our study, we analyzed the distribution of Th17 cells in the peripheral blood of acute leukemia patients and showed that the pretreatment level of Th17 cells was higher in patients than in healthy volunteers and in patients with favorable response to induction therapy than non-responders. Muranski et al. [23] found that tumor-specific Th17-polarized cells could eradicate large established melanomas in mice. That is, Th17 cells exerted an anti-tumor effect, which was also proved in other solid tumors [7, 24, 25]. In light of these results, elevation in Th17 cells in our acute leukemia patients may be explained as a protective reaction of the immune system in certain stages of the disease.

We have further demonstrated that the increased circulating Th17 cells in untreated patients were reduced when those patients achieved CR after chemotherapy, suggesting that circulating Th17 cells are related to tumor burden and could be valuable for monitoring response to chemotherapy in acute leukemia patients. A previous study has also described increased frequencies of circulating Th17 cells in patients with untreated AML with normalization when patients achieved complete remission after chemotherapy [17]. Interestingly, elevated levels of Th17 cells have been also found in patients with favorable response to therapy in some tumors [26]. In contrast to our results, Ersvaer et al. [18] have demonstrated normal Th17 levels for patients with untreated AML which did not differ from healthy controls. However, controls in that study were younger than untreated patients. Before statistical comparisons and if any age-dependent difference among the controls was detected, Ersvaer and his colleagues used to compare untreated patients with those controls being above 50 years of age (*n* = 15), which may be not adequate to show a difference between AML patients and controls.

IL-17 is the hallmark effector cytokine produced by Th17 cells [16]. The biological function of Th17 cells is closely associated with its secreted IL-17 [18]. Among Th17-associated cytokines, IL-21 appears to promote Th17 differentiation and serves as an autocrine regulator of IL-17 production [15]. Using ELISA, we found that serum levels of IL-17 and IL-21 in untreated acute leukemia patients were significantly higher than in controls and correlated positively with levels of circulating Th17 cells. Therefore, we believe that tumor-associated Th17 cells have the ability to influence immune responses and produce anti-tumor effect through the action of any one or a combination of their related cytokines.

In regard to survival, we documented, for the first time, a positive correlation between circulating Th17 cells and survival in this group of acute leukemia patients. Even after controlling for other prognostic factors, Th17 cells remained a negative predictor of death hazard, suggesting that Th17 cells exert direct or indirect anti-tumor effect in acute leukemia and may be a prognostic indicator for this group of patients.

Lastly, we assessed the correlation between serum IL-17 level and the prognosis of the same group of patients. As might be expected, higher IL-17 serum level was also correlated with longer survival, which is consistent with previous demonstration that IL-17 and IL-6 and IL-1β, which promote human Th17 cell differentiation, are members of clusters of cytokines that correlate with better prognosis in CLL [27].

Of note, elevated levels of Th17 cells have also been reported in CLL patients and were proved to favorably modify the clinical course of this group of patients, regardless of prognostic or genetic subgroup [28]. Along this line, Kryczek et al. [7] have detected that levels of Th17 cells were lower in the ascites of advanced ovarian cancer patients, which may have positive effect on prognosis of patients. Hence, their data provide evidence that Th17 cells may contribute to protective tumor immunity in humans with advanced tumors. In contrast, our results were discrepant with previous data on pancreatic cancer, which revealed that median survival time was shorter in patients with high level of Th17 cells [29]. However, this discrepancy may be due to the different biological characteristics among different tumor types [11], and the possibility that the function of Th17 cells may vary according to different cancer cause, type, and location, as well as stage of cancer.

Demonstration of the effect of IL-21 in acute leukemia cell lines and acute leukemia patients remains unproven. Previously published reports indicated that IL-21, secreted by Th17 cells, promotes fludarabine- and rituximab-mediated direct apoptosis of CLL cells [30]. Brenne et al. [31] found that IL-21 induced proliferation and inhibited apoptosis in the IL-6-dependent human myeloma cell lines. In AML, Li et al. [16] detected the IL-21 levels in plasma samples taken from 24 AML patients before chemotherapy, 20 AML patients with CR, and 30 health adults, and found no significant differences between groups. Our results detected positive correlation between IL-21 and survival in ALL, which raise the possibility that IL-21 has anti-leukemic effect in this group of patients. In contrast, we failed to prove correlation between pretreatment IL-21 serum level and survival in AML. We further identified significantly elevated serum IL-21 levels in ALL than AML patients. Therefore, we assume that T cell immune reaction to leukemia appears to be much higher in ALL than in AML. However, more research on large number of patients will be needed to confirm these data.

In conclusion, we explored Th17 cells in acute leukemia patients, as a valuable marker for monitoring patients after chemotherapy, and documented their association with a more favorable outcome. Our data provides clinical evidence linking Th17 cells to immune protection in acute leukemia and opens a new avenue in the study of tumor immunotherapy based on promoting Th17 cell population.
